# Interrater reliability of clinical tests to evaluate scapulothoracic motion

**DOI:** 10.1186/1471-2474-14-315

**Published:** 2013-11-05

**Authors:** Evelyn Baertschi, Jaap Swanenburg, Florian Brunner, Jan Kool

**Affiliations:** 1 Department of Physiotherapy, Uniklinik Balgrist, Forchstrasse 340, 8008 Zurich, Switzerland; 2 Department of Physical Medicine and Rheumatology, Uniklinik Balgrist, Zurich, Switzerland; 3 Department of Health, Institute of Physiotherapy, Zurich University of Applied Sciences, Winterthur, Switzerland

## Abstract

**Background:**

Decreased scapulothoracic motion has been associated with various pathologies of the shoulder. Reliable and simple assessment methods of scapular mobility are, however lacking. The aim of this study was to evaluate the interrater reliability of four clinical tests to assess scapulothoracic motion in patients with a slightly restricted shoulder flexion.

**Methods:**

A total of nineteen patients with a symptomatic slight restriction of shoulder flexion and twenty asymptomatic subjects were evaluated. The investigation consisted of four palpatory tests to assess scapulothoracic motion. A two-level rating scale (positive, negative) was utilised. Interrater reliability was evaluated using kappa coefficients.

**Results:**

We found substantial to almost perfect (Kappa = 0.63-0.4) interrater reliability for the four tests.

**Conclusion:**

Our study demonstrates that the four mobility tests of the shoulder are a reliable and simple instrument to assess patients with a slightly restricted shoulder flexion. Future studies should be conducted to evaluate the validity of these tests and to establish their clinical usefulness.

## Background

Scapular movement plays a key role in shoulder and arm function. The scapula acts as a stable base for optimal muscle activation and as a transfer link for developed forces in the kinetic chain [1]. Shoulder pain and several pathologies, such as shoulder impingement, rotator cuff tendinopathy, rotator cuff tears, glenohumeral instability, adhesive capsulitis and stiff shoulders, are associated with alteration in scapular kinematics [2-4]. Therefore, the evaluation of scapular kinematics should form a part of clinical shoulder joint examination.

Previous assessment methods of scapular motion focussed on visual observation [5-8]. In these studies winging, or dysrhythmia of scapular motion during shoulder flexion or abduction was rated. Decreased scapular motion was not an assessment criterion. However, a correlation between decreased scapular upward rotation and glenohumeral instability has been demonstrated [3,9,10]. Furthermore, it is generally believed, that reductions in scapular upward rotation and posterior tilt during arm elevation could contribute to subacromial impingement by reducing the available subacromial space [3,5,11-14]. Contrary to this, another investigation showed an increase of the subacromial space with reduction of scapular upward rotation [15]. Conflicting results are also found in the direction of scapular motion alterations in shoulder impingement [3]. The numerous methods of recording scapular motion, the variation in movement patterns in subjects and the investigation of scapular motion in different static positions or variations of shoulder elevation, might have contributed to this variability of findings.

By using x-ray cinematographic analyses, Stenvers and radiologists from the Martini hospital in Groningen associated decreased scapular upward rotation, posterior tilt and external rotation with a slight restriction of shoulder flexion (± 150°) and altered motion of the clavicle and the cervicothoracic junction [16]. Stenvers further noted that in these patients glenohumeral range of motion in flexion and abduction is not, or only insignificantly restricted. He described this clinical pattern as, “the slightly restricted shoulder”. He observed the following disorders in patients with a slightly restricted shoulder: subacromial impingement, coracoclavicular compression, excessive torsion in the acromioclavicular joint and a tendency towards glenohumeral instability [17].

To identify patients with decreased scapular motion and to allow a more targeted treatment of these patients, physiotherapists require a reliable and easily performed clinical assessment method.

One measurement instrument with good to excellent intrarater reliability in the assessment of scapular upward rotation in different positions of shoulder abduction, is the inclinometer [18].

This method would be well suited to documenting progress during therapy. However, in order to select patients for targeted treatment, data both from healthy subjects and for interrater reliability, are not available. Furthermore, only shoulder abduction and not flexion has been investigated. Asymmetry of scapular motion, however, is more evident in flexion than in abduction [6].

Stenvers and Overbeek [16] described four palpatory mobility tests of the shoulder to identify patients with a slightly restricted shoulder. These tests are easy to apply in clinical practice and include evaluation of scapular, clavicular and cervicothoracic motion. To our knowledge, this is the first study to investigate the reliability of palpatory tests on scapulothoracic motion.

The aim of this study was to evaluate the interrater reliability of four tests in the assessment of scapular motion during shoulder flexion.

## Methods

### Participants

A total of thirty nine participants were recruited from the Physiotherapy Department of the Balgrist University Hospital in Zurich, Switzerland; nineteen patients with symptomatic slight restriction of shoulder flexion and twenty controls with no shoulder symptoms. Sample size calculation was based on identifying a moderate strength of agreement (Kappa > 0.4) at a significance level of 0.01 and a power of 80% [19].

Patients were included if they presented with shoulder complaints and passive shoulder flexion of at least 130 degrees in a standing position. Thus, patients with a predominantly glenohumeral restriction were excluded. Controls had no shoulder complaints or any other complaints which might have had an impact on shoulder function. Participants were excluded if they had had shoulder or spinal surgery less than six weeks previously, or suffered from neurological disorders or scoliosis.

Approval was obtained from the Cantonal Ethics Committee of Zurich (KEK-ZH: 2011–0387). Written informed consent of each study participant was obtained.

### Examination

**Figure 1:**
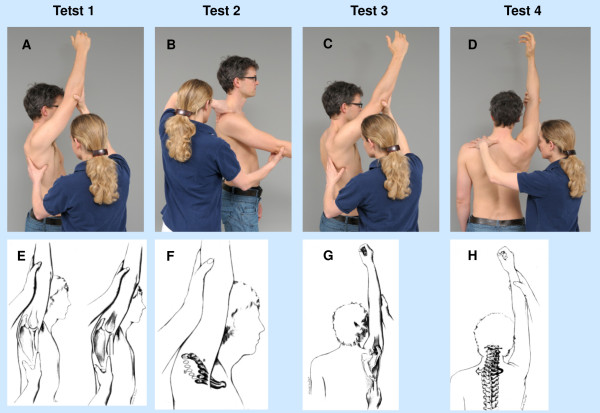
**Test 1–4. A-D** Normal performance of the mobility tests of the shoulder. **E-H** Normal and abnormal performance **E** Position of the most lateral scapular point at the end of flexion (left: normal, right: slightly restricted shoulder). **F** Clavicular motion during flexion in normal shoulder movement (bold) and in a slightly restricted shoulder (not bold). **G** Scapular posterior tilting during the last phase of flexion (normal shoulder movement). **H** Movement of the cervicothoracic junction during the last phase of flexion (normal shoulder movement). **E-H** With kind permission of J.D. Stenvers (Stenvers, van Woerden & Kingma, 2011). **A-D** With the consent of the individuals shown in these images.

The examination consisted of the mobility tests of the shoulder, as described by Stenvers and Overbeek [16] (Figure 1):

•Test 1: Scapular axillary hair test at the end of flexion.

•Test 2: Clavicular movement during the first 60 degrees of flexion.

•Test 3: Scapular posterior tilting during the last phase of flexion.

•Test 4: Movement of the cervicothoracic junction during the last phase of flexion.

Decreased scapular upward rotation, posterior tilt and external rotation, combined with altered motion of the clavicle and the cervicothoracic junction, was defined as a restriction of scapular motion. Flexion was defined as lifting the arm in the sagittal plane. Tests were performed in a standardised order. Participants stood upright, legs one foot-width apart and facing straight ahead. The symptomatic shoulder or alternately the right and left shoulder (controls) was evaluated. Each test was rated either positive (restriction of scapular motion is present) or negative (scapular motion is normal).

#### Test 1: Scapular axillary hair test at the end of flexion

Stenvers [20] and de Wijer [21] suggested the dorsal axillary hair borderline as a reliable measurement point for the position of the scapula at the end of flexion.

The investigator passively moves the patient’s arm to the end of shoulder flexion. In this position the distance from the most lateral scapular point (crista margo lateralis inferior) and the vertical extension of the dorsal axillary hair borderline is determined.

*Negative*: If the most lateral scapular point is in the vertical extension of the dorsal axillary hair borderline, the test was rated negative.

*Positive*: If the determined distance was one finger width or more, the test was rated positive.

#### Test 2: Clavicular movement during the first 60 degrees of flexion

During normal shoulder flexion the clavicle moves anteriorly during the first 60 degrees. As a result, space in supraclavicular fossa increases. Subsequently, the clavicle continues to move cranially and posteriorly and, finally, caudally (ellipsoid path) [22]. Other studies have described posterior rotation, retraction and minimal elevation of the clavicle during normal elevation of the arm [23,24].

The investigator passively moves the patient’s arm to approximately 60 degrees of shoulder flexion. By means of palpation the clavicular motion is simultaneously assessed.

A decreased rotation can be observed in addition to the following pattern: at the beginning of the movement the clavicle moves posteriorly, followed later by movement in a dorsocranial direction (Figure 1F) [22]. Thus, the palpating finger is pushed out of the supraclavicular fossa.

A different movement pattern of the clavicle was described in another study that analysed three-dimensional motion of the clavicle in symptomatic shoulder patients [25]. The authors found a greater clavicular elevation at 90 and 120 degrees of shoulder flexion in subacromial impingement compared to healthy subjects. The differences between groups below 90 degrees of flexion were statistically not significant.

*Negative*: If the clavicle “stood still”, or a small fosse was formed for the palpating finger, the test was rated negative.

*Positive*: The test was rated positive if the clavicle pushed the palpating finger cranially out of the supraclavicular fossa.

#### Test 3: Scapular posterior tilting during the last phase of flexion

The investigator passively moves the patient’s arm to the end of shoulder flexion. The scapula is palpated simultaneously.

*Negative*: If the inferior angle of the scapula moved caudal and anterior at the end of shoulder flexion, the test was rated negative.

*Positive*: If this movement could not be felt, the test was rated positive.

#### Test 4: Movement of the cervicothoracic junction during the last phase of flexion

During shoulder movement not only the scapulothoracic, the acromioclavicular, sternoclavicular and glenohumeral joints are involved, but also the cervical and thoracic spine. At the normal end of range flexion of the shoulder the cervicothoracic junction moves in extension, contralateral lateral flexion and ipsilateral rotation. There is a significant correlation between restriction of scapular motion and restricted movement of cervicothoracic junction [20,26].

The investigator passively moves the patient’s arm to approximately 30 degrees before the end of shoulder flexion. During passive end of range flexion (the last 30°) the spinal segments C7-T4 are palpated.

*Negative:* If an ipsilateral rotation of the spinous processes from C7-T4 could be palpated, the test was rated negative.

*Positive*: If this movement could not be palpated, the test was rated positive.

### Procedure

**Figure 2:**
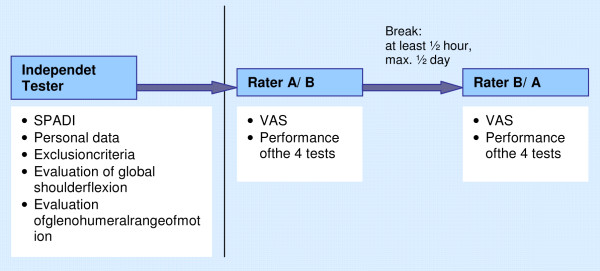
Procedure.

The study procedure is summarized in Figure 2. Prior to the testing sessions, an experienced, independent physiotherapist specializing in musculoskeletal therapy reviewed the exclusion criteria, conducted a brief survey (Table 1) and determined glenohumeral and cervical range of motion. Glenohumeral range of motion of the test shoulder was assessed using a goniometer. Flexion, abduction and external rotation in 0 degrees of glenohumeral abduction were assessed in the seated position. Internal and external rotation in 90 degrees of glenohumeral abduction were assessed in prone position. Goniometric measurement of glenohumeral joint range is more reliable than visual estimation [27]. Active-assistive cervical range of motion was assessed in the seated position. Cervical flexion and extension was measured with an inclinometer. This instrument has been recommended as reliable [28]. Cervical rotation was visually estimated. This method showed substantial to perfect intra- and interrater reliability of cervical range of motion [29]. Patients completed the Shoulder Pain and Disability Index (SPADI). The SPADI is a subjective measurement instrument for assessing patients with shoulder disorders. It contains a five-item subscale that measures pain and an eight-item subscale for disability. Each item is scored on a visual analogue scale [30]. This questionnaire was translated into German and reliability and validity were confirmed [31].

**Table 1:** Subject characteristics

	**Patients**	**Controls**
Number	19	20
Female/male	9/10	15/5
Mean age, yrs (SD, range)	46 (15, 21–66)	32 (9, 20–55)
Dominant hand: right/left	19/0	17/3
Shoulder tested: right/left	11/8	10/10
Mean symptom duration, mths (SD, range)	26 (29, 1–120)	---
SPADI ^a)^; mean (SD, range)		
Overall (0–100)	17 (12, 4–48)	---
Pain (0–100)	24 (17, 6–64)	---
Disability (0–100)	12 (10, 0–39)	---
Diagnoses (number)		
Rotator cuff repair	4	---
SLAP^b)^ lesion	2	---
Glenohumeral instability	2	---
Osteosynthesis (humerus, clavicle)	2	---
Shoulder impingement	1	---
Rotator cuff tear	1	---
Capsulotomy	1	---
Nonspecific shoulder pain	6	---
Other complaints (number)	9 (47%)	6 (30%)
Cervical spine	5	3
Lumbar spine	4	4
Hand	4	1
Ellbow	3	---
Head	2	---
Thoracic spine	---	1
Sports on a regular basis (number)	17 (89%)	18 (90%)
Gym	4	7
Jogging	6	6
Skiing, wintersport	4	4
Biking	3	5
Swimming	3	2
Horse-riding	1	3
Volleyball/tennis	3	2
Climbing	---	4
Golf	2	---
Other	7	4

a) SPADI = Shoulder Pain and Disability Index; b) SLAP = Superior Labrum Anterior to Posterior.

Two experienced physiotherapists specializing in musculoskeletal therapy performed the mobility tests of the shoulder. They received further training from J.D. Stenvers at the training centre for neck, shoulder and arm disorders (NSA). Prior to the experiment they underwent an additional training in order to be familiarised with the test performance and rating criteria. Raters were blinded to the patients’ diagnoses. The order of the four tests for each participant was the same for both testing sessions. Each session did not last longer than five minutes. The order of the two raters was changed after each participant. There was a break of at least thirty minutes to maximum half a day between the two testing sessions to minimise testing bias between the two investigations. Participants did not receive any therapeutic treatment for the shoulder during the break. To verify whether pain intensity was comparable at the start of both testing sessions, raters recorded the participant’s current pain intensity on a visual analogue scale (VAS: 0–10) at the beginning of each testing session.

### Statistical analysis

A paired samples t-test was conducted to compare pain intensity at the beginning of the two testing sessions.

Interrater reliability was evaluated using kappa coefficients [32] and percentage agreement. However, where the prevalence is not around 50% to a particular outcome between raters or in the presence of bias, kappa can be affected [33]. Therefore, prevalence and bias indices, as well as a prevalence-adjusted-bias-adjusted kappa (PABAK) coefficient were calculated [19]. The classification system proposed by Landis and Koch [34] was used to determine the level of reliability for Kappa and PABAK: <0: poor, 0.00-0.20: slight, 0.21-0.40: fair, 0.41-0.60: moderate, 0.61-0.80: substantial, 0.81-1.00: almost perfect. A kappa coefficient of over 0.40 for clinical tests was considered to be acceptable in comparable studies [8,22,35,36]. Accordingly in this study, kappa coefficients of over 0.40 were judged as being satisfactory.

Statistical analysis was performed using IBM-SPSS 17 (SPSS, Inc., Chicago, USA).

## Results

The participants’ characteristics are presented in Table 1. Patients presented with eight different clinical diagnoses. Median symptom duration was two years. Pain and disability in daily living were rather low (Shoulder Pain and Disability Index: 17/ 100). Head, spinal, elbow or hand disorders had been experienced by nine patients (47%) and six controls (30%) during the previous twelve months. Approximately 90% of participants practiced sports on a regular basis.

Range of motion was significantly restricted in the patient group compared with controls (Table 2). The findings of the patient group can be clinically described as an end of range limitation.

**Table 2:** Glenohumeral and cervical spine range of motion

	**Patients**	**Controls**
Mean glenohumeral range of motion, degree (SD, range)		
Flexion	85 (8, 70–95)	91 (2, 90–95)
Abduction	88 (5, 80–95)	93 (4, 85–100)
External rotation in 0° abduction	40 (15, 15–70)	60 (12, 30–80)
External rotation in 90° abduction	79 (17, 45–100)	94 (5, 80–100)
Internal rotation in 90° abduction	36 (17, 10–75)	53 (14, 20–80)
Mean cervical range of motion, degree: (SD, range)		
Flexion	58 (7, 45–70)	61 (11, 45–80)
Extension	56 (11, 35–80)	74 (10, 55–90)
Rotation to the right	72 (7, 60–85)	81 (9, 60–90)
Rotation to the left	71 (10, 50–85)	81 (8, 65–90)

Mean pain intensity at the start of the session was 1.3/ 10 for rater A and 1.4/ 10 for rater B, indicating no significant difference in pain level at the start of each testing session (paired t-test, p = 0.21).

The frequency of positive rating was 63-89% for patients and 5-30% for controls.

Table 3 gives an overview of the attained results. The percentage of agreement varied between 82-92%. For the overall results of patients and controls, three out of four tests showed substantial interrater reliability (Kappa: 0.61-0.80) and one test had a kappa value >0.81 (almost perfect). Prevalence and bias indices were low and the value of the PABAK was no different to the kappa. In separate evaluations of the two groups, kappa values were interpreted as fair, moderate or substantial (Kappa: 0.21-0.80). The prevalence index was high and the bias index was low. The PABAK value was higher than the unadjusted kappa and was interpreted as moderate (PABAK: 0.41-0.60) to almost perfect (PABAK: 0.81-1.00).

**Table 3:** Reliability of the moblity tests of the shoulder

	**Percent agreement**	**Kappa (κ) 95% CI**	**PABAK 95% CI**	**Bias index**	**Prevalance index**
**Test 1 **Patients	17/19 (89%)	0.60 (0.11, 1.00)	0.79 (0.51, 1.00)	0.00	0.68
Controls	18/20 (90%)	0.74 (0.40, 1.00)	0.80 (0.54, 1.00)	0.10	0.50
Total	35/39 (90%)	**0.79** (0.60, 0.98)	0.79 (0.60, 0.99)	0.05	0.07
**Test 2 **Patients	15/19 (79%)	0.52 (0.12, 0.92)	0.57 (0.21, 0.95)	0.10	0.36
Controls	17/20 (85%)	0.35 (0.00, 0.86)	0.70 (0.39, 1.00)	0.15	0.85
Total	32/39 (82%)	**0.63** (0.39, 0.87)	0.64 (0.40, 0.88)	0.12	0.20
**Test 3** Patients	17/19 (89%)	0.60 (0.11, 1.00)	0.79 (0.51, 1.00)	0.00	0.68
Controls	17/20 (85%)	0.57 (0.14, 1.00)	0.70 (0.39, 1.00)	0.05	0.55
Total	34/39 (87%)	**0.74** (0.53, 0.95)	0.74 (0.53, 0.95)	0.02	0.05
**Test 4** Patients	18/19 (95%)	0.64 (0.00, 1.00)	0.90 (0.70, 1.00)	0.05	0.84
Controls	18/20 (90%)	0.74 (0.40, 1.00)	0.80 (0.54, 1.00)	0.10	0.50
Total	36/39 (92%)	**0.84** (0.67, 1.00)	0.85 (0.68, 1.00)	0.02	0.15

Bold: Kappa values of the total sample size.

## Discussion

To our knowledge, this is the first study to investigate the reliability of palpatory tests of scapulothoracic motion. We found substantial to almost perfect reliability for the four mobility tests of the shoulder in patients with a slightly restricted shoulder and asymptomatic participants when performed by two experienced physiotherapists.

Looking at the kappa values of patients and controls separately, the results were fair to substantial. These differences can be explained by the smaller sample sizes and high prevalence indices. A prevalence effect exists when the proportion of agreements on the positive classification differs from that of the negative classification [19]. The number of positive tests was very high in patients and very low in controls. Both the high prevalence indices and the smaller sample size affected the confidence interval [37]. To examine the effects of prevalence on kappa values and confidence interval, PABAK values were calculated. These values were higher than the unadjusted kappa values. However, for interrater agreement, it is the result from the total sample size that is of particular interest. The bias indices were low over all, indicating that systematic bias of an examiner did not influence the results.

Other studies investigating scapular motion have obtained lower interrater reliability than this study [6,7]. Uhl et al. [6] and McClure et al. [7] assessed scapular motion through visual observation and not by use of palpation. The use of palpatory tests might have contributed to the higher reliability. McClure et al. [7] used a 3-level scale (normal, subtle, obvious change of motion) to rate scapular motion. The use of only two levels (positive, negative) may also account for the higher kappa values observed in this study.

Stenvers et al. [20] argue that the four tests are of little value on their own. They propose a diagnosis of a restriction of scapular movement only when at least three of the four tests are positive. This clinical application of the four analysed tests has no negative influence on interrater agreement. If the scapulothoracic motion of the thirty-nine participants is assessed as Stenvers has suggested, the interrater reliability is substantial (Kappa = 0.74). Whether this “overall assessment “of the four tests achieves a greater specificity and sensitivity than the individual tests cannot be evaluated from this study. A reference test, or gold standard, is not available.

Several factors should be considered when interpreting the level of reliability found in this study. Since the four tests were always conducted consecutively, the rater’s decision may have been influenced by the outcome of preceding tests. However, for decision making in clinical practice, it is standard to consider the results of all four tests rather than taking into account just one single test.

Another limitation of this study is that, since controls had no limitation of shoulder movement, raters could not be blinded to the participants’ group. Thus, their judgement may have been influenced by their expectations. Blinding was also difficult due to the evaluation of pain at the beginning of every testing session.

Before generalising the results of this study, it should be considered that the two raters were experienced physiotherapists with a specialisation in musculoskeletal therapy and had also received further training in the treatment of neck, shoulder and arm disorders. Future research is required to investigate whether satisfactory levels of reliability can also be achieved using less experienced clinicians without specific training.

## Conclusion

Our study demonstrates that the four mobility tests of the shoulder are a reliable and simple instrument in the assessment of patients with a slightly restricted shoulder flexion. Nevertheless, validity has to be investigated before the clinical utilisation of these tests can be approved, e.g. comparing the four mobility tests of the shoulder with electromagnetic kinematic testing.

Moreover, further studies are needed to show correlations between restricted scapular motion and shoulder pathologies. Additionally, the effect of specific treatment of patients with restricted scapular motion would be of great interest.

## Competing interests

The authors declare that they have no competing interests.

## Authors’ contributions

EB designed the study, collected data and was the main writer of the paper. JS performed the statistical analyses and critically reviewed the protocol and the manuscript. FB critically reviewed the manuscript. JK participated in the study design and critically reviewed the manuscript. All authors read and approved the final manuscript.

## Pre-publication history

The pre-publication history for this paper can be accessed here:

http://www.biomedcentral.com/1471-2474/14/315/prepub
